# Supplementary material for: A Novel Mouse Model of Combined Hepatocellular-Cholangiocarcinoma Induced by Diethylnitrosamine and Loss of Ppp2r5d
Source: Cancers (Basel). 2023 Aug 21;15(16):4193. doi: 10.3390/cancers15164193 (PMC10453342; doi:10.3390/cancers15164193)
Supplement: Supplementary file 1 [file cancers-15-04193-s001.zip › cancers-2533214-supplementary.pdf]

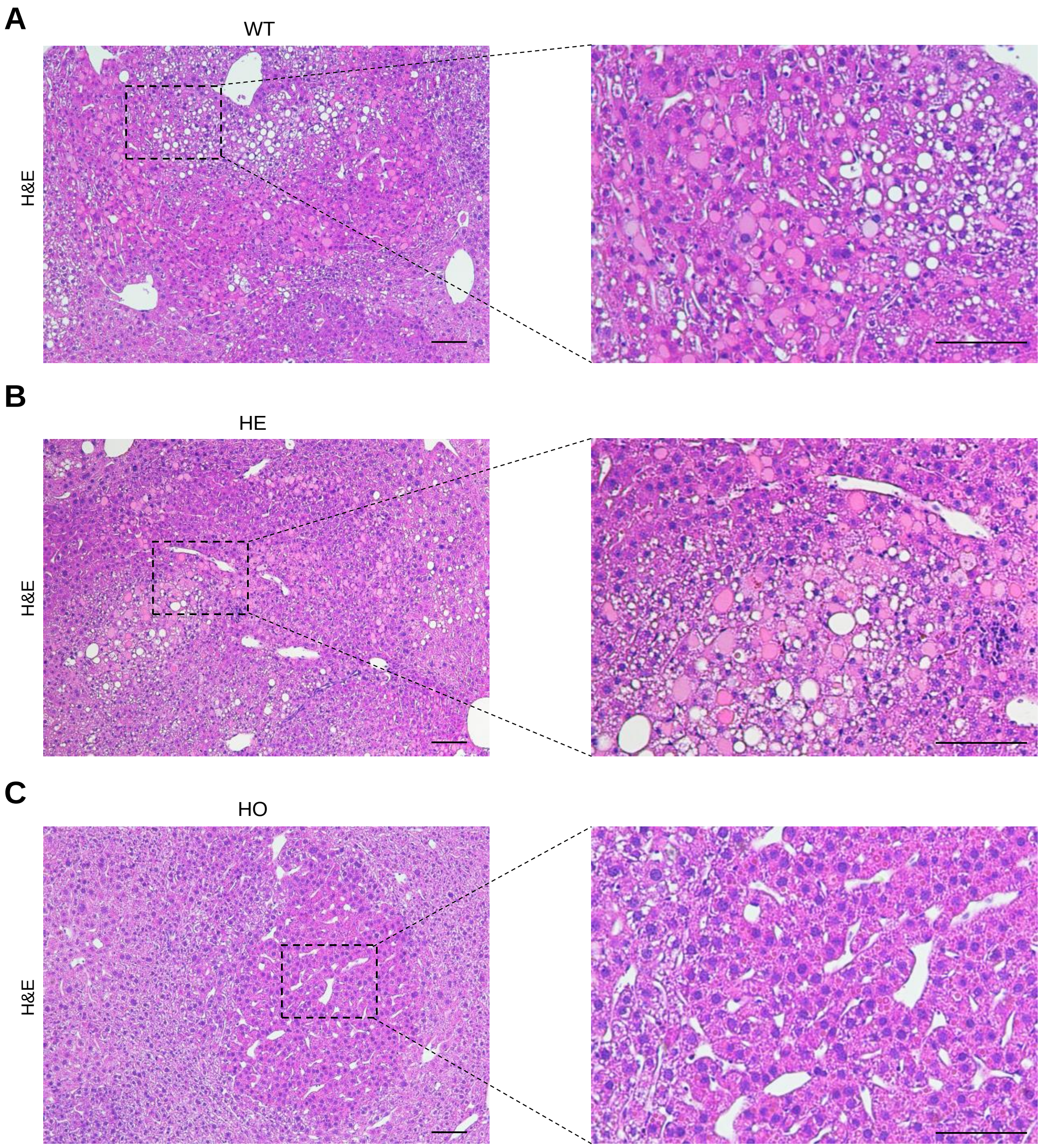

Figure S1. Histologic liver analysis of WT, HE, and HO *Ppp2r5d* KO mice at 9 months post-DEN treatment. (A) Representative image of a liver section from a WT mouse. The panel on the right is an amplification of the area delineated with dotted lines showing the clear ballooned cells with coarse inclusion bodies and steatosis **(B)** Representative image of a liver section from a HE mouse. The panel on the right is an amplification of the area delineated with dotted lines showing the nodule-in-nodule with steatosis **(C)** Representative image of a liver section from a HO mouse. The panel on the right is an amplification of the area delineated with dotted lines showing a nodule with broad trabecula growing pattern**.**

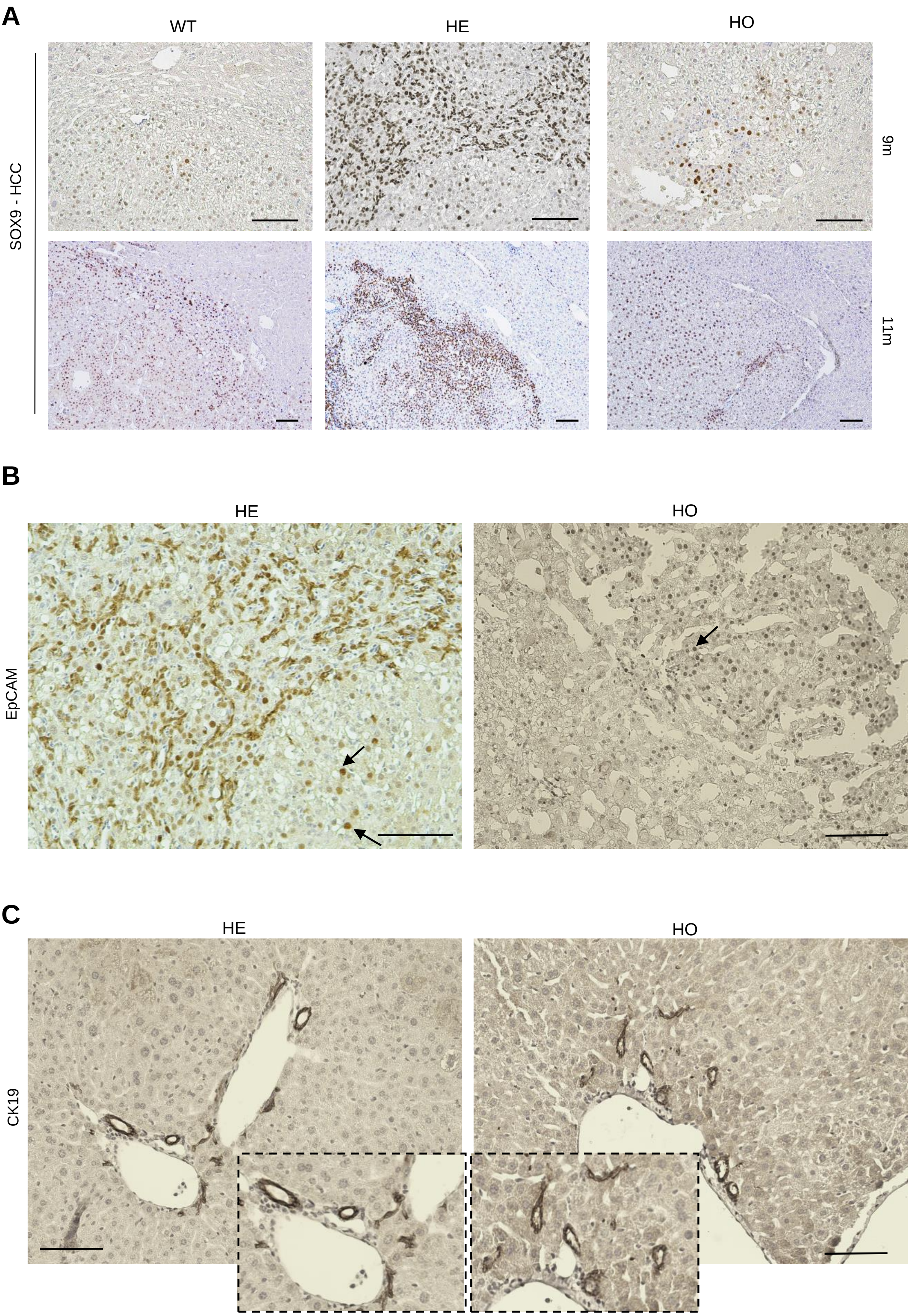

Figure S2. Immunohistochemical analysis of HCC tumors from WT, HE, and HO *Ppp2r5d* KO mice at 6, 9, and 11 months post-DEN treatment. (A) Representative images of liver sections of WT, HE, and HO *Ppp2r5d* KO mice stained for SOX9. Hepatocytes from some HCC tumors at 9 and 11 months post-DEN treatment were positive for SOX9, indicative of a less differentiated state **(B)** Representative image of liver sections of HE, and *Ppp2r5d* KO mice stained for EpCAM. In some occasions hepatocytes in HCC tumors were positive for Epcam, indicative of an intermediate phenotype between hepatocytes and bile ducts **(C)** Representative image of liver sections of HE, HO *Ppp2r5d* KO mice stained for CK19. Some mice at 6 months post-DEN treatment developed ductular reaction. Scale bar represents 100µm.

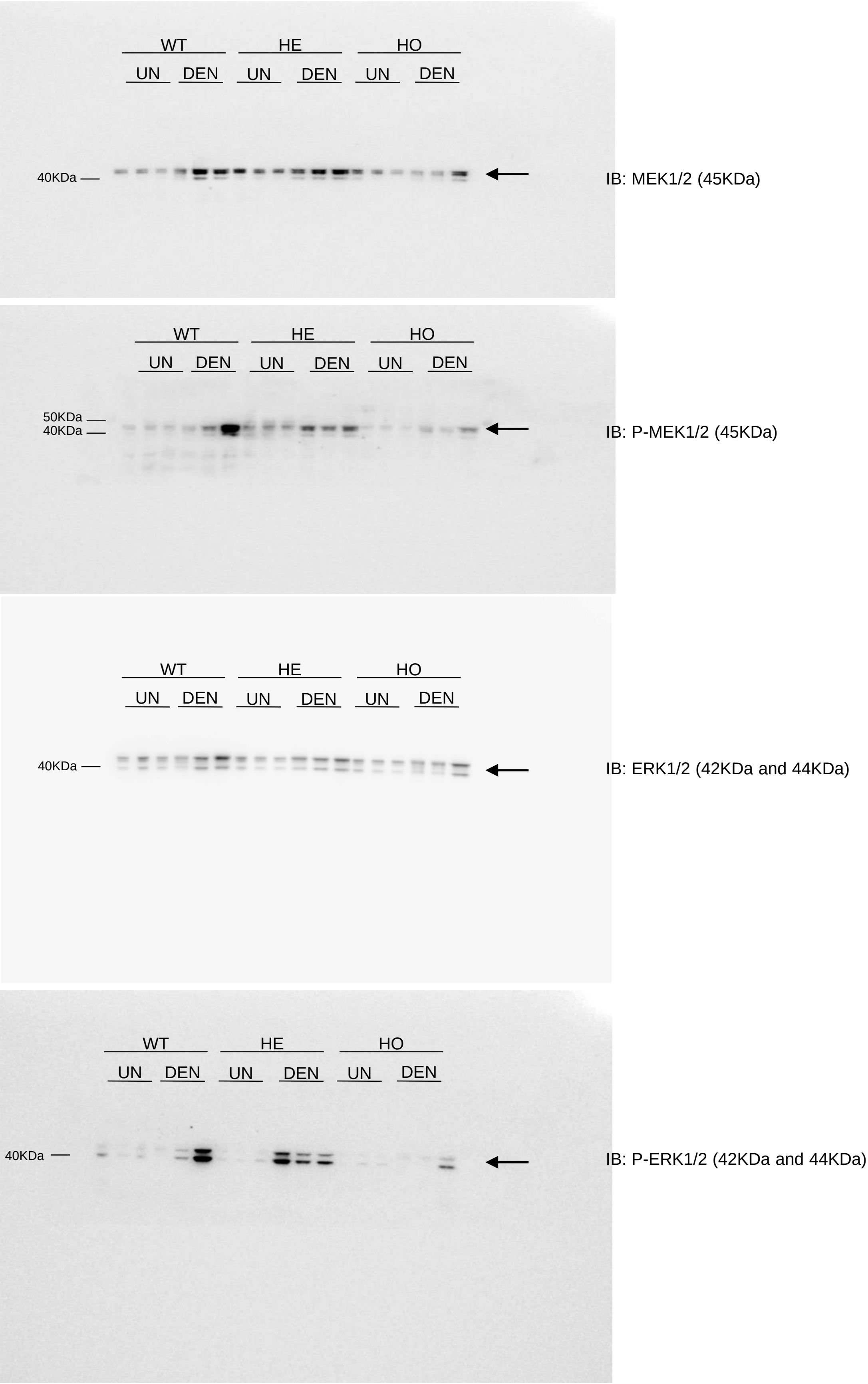

Figure S3. Uncropped blots from Figure 6A. Total MEK1/2, P(Ser2177/221) MEK1/2, total ERK1/2, P(Thr202/Tyr204) ERK1/2.

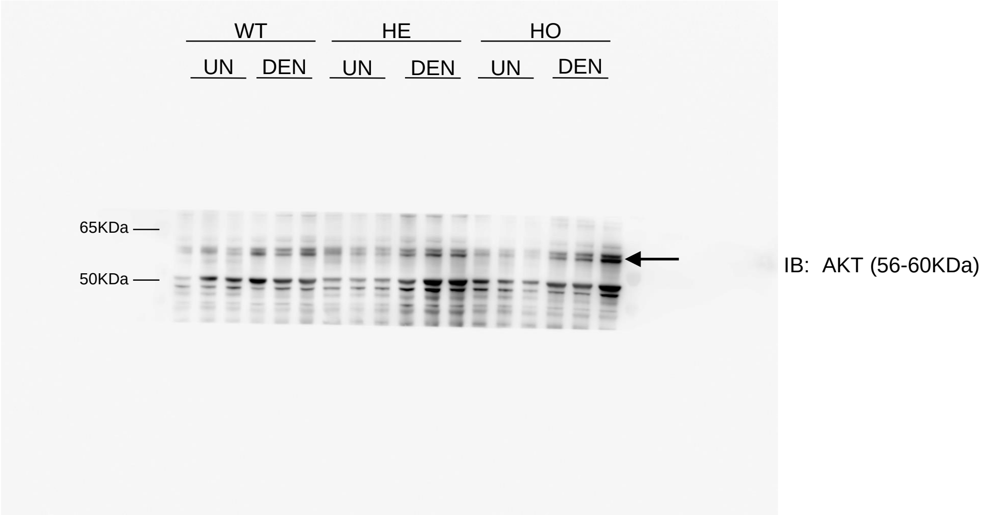

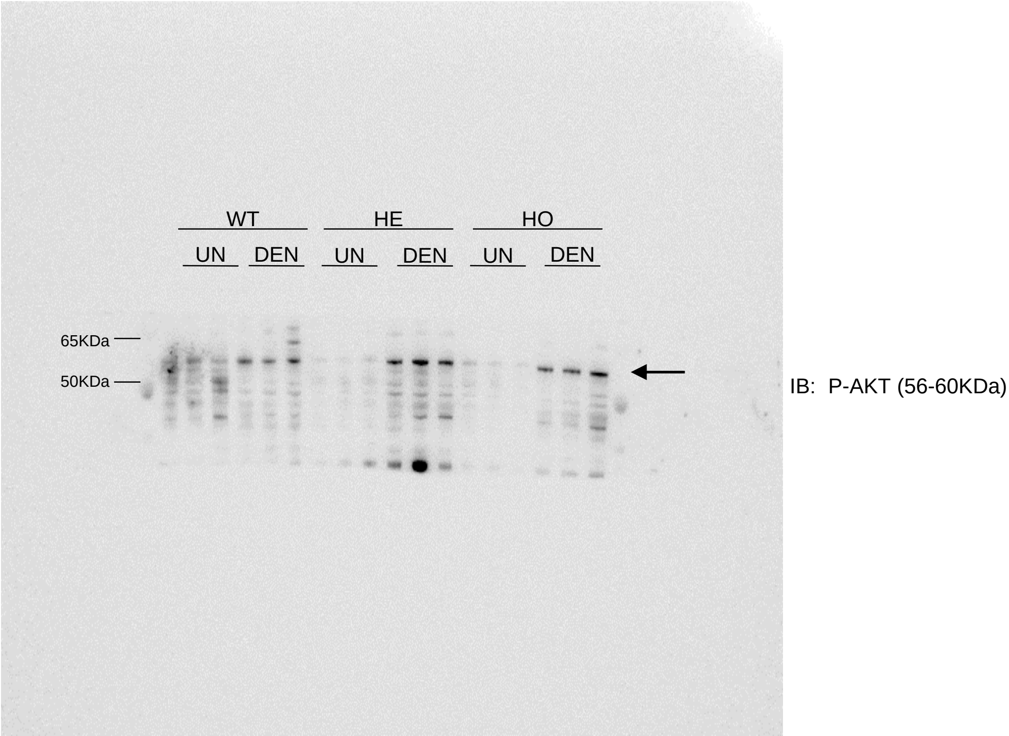

Figure S4. Uncropped blots from Figure 6B. Total AKT, P(Thr308) AKT.

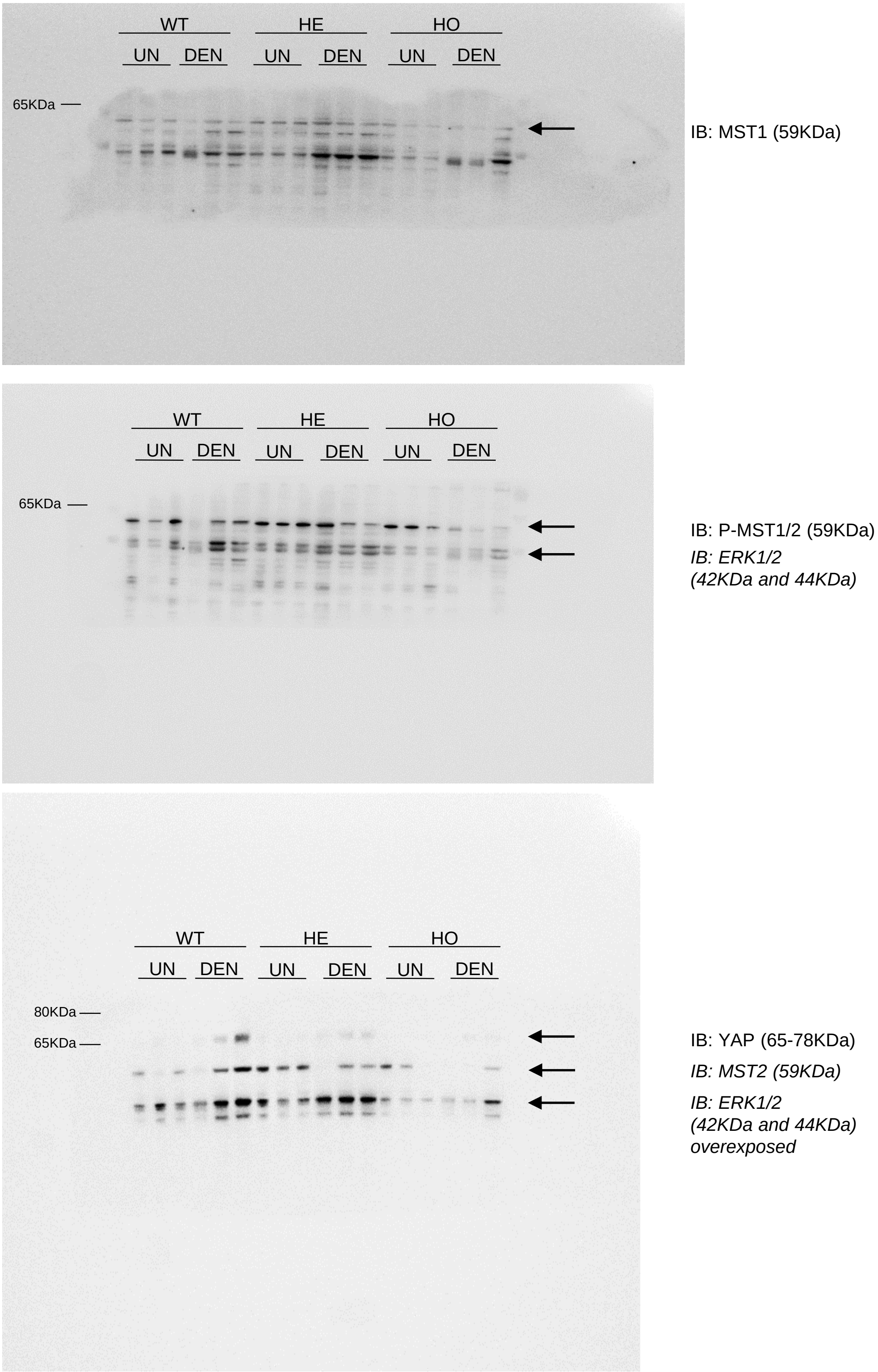

Figure S5. Uncropped blots from Figure 6C. Total MST1, P(Thr183/Thr180) MST1/2, total YAP. Bands corresponding to previous incubations are indicated in italics.

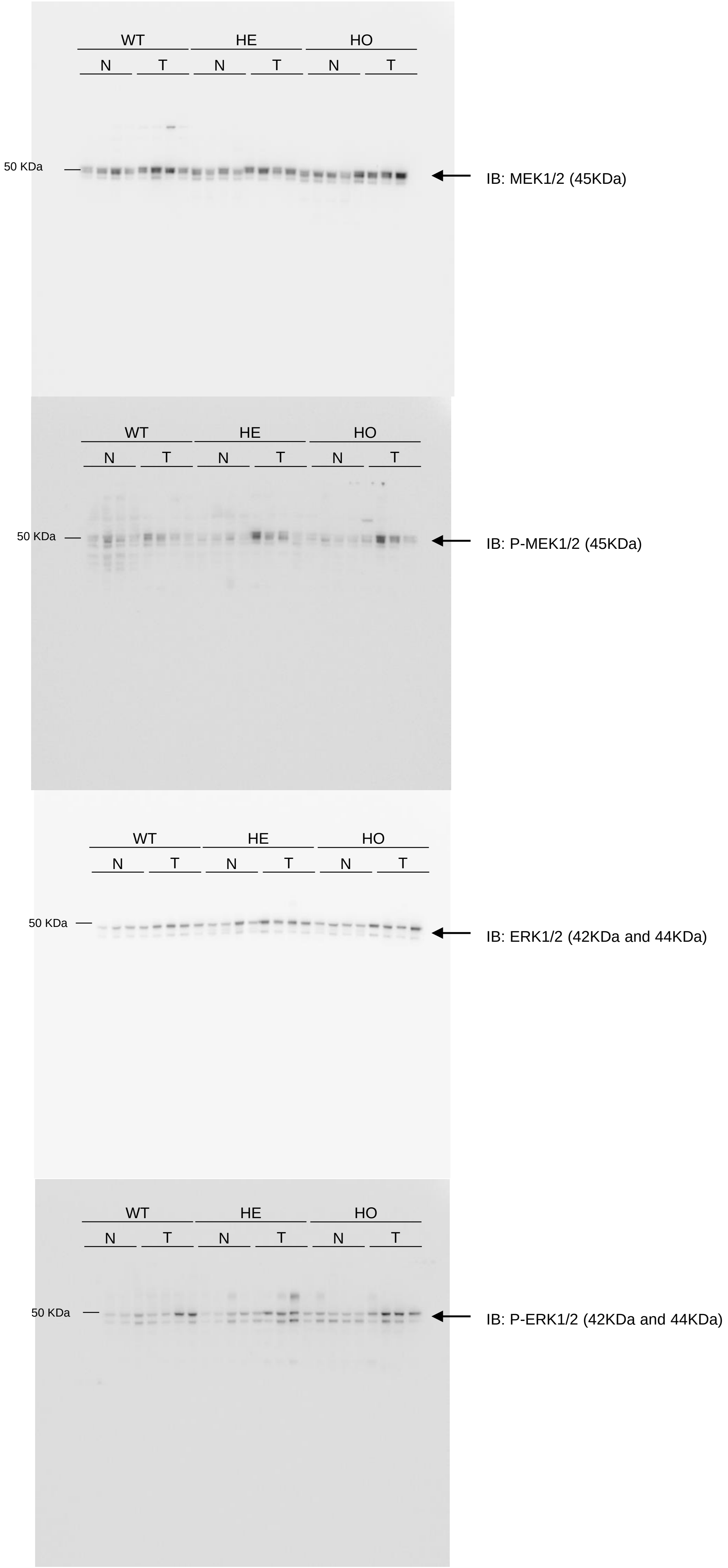

Figure S6. Uncropped blots from Figure 7A. Total MEK1/2, P(Ser2177/221) MEK1/2, total ERK, and P(Thr202/Tyr204) ERK1/2.

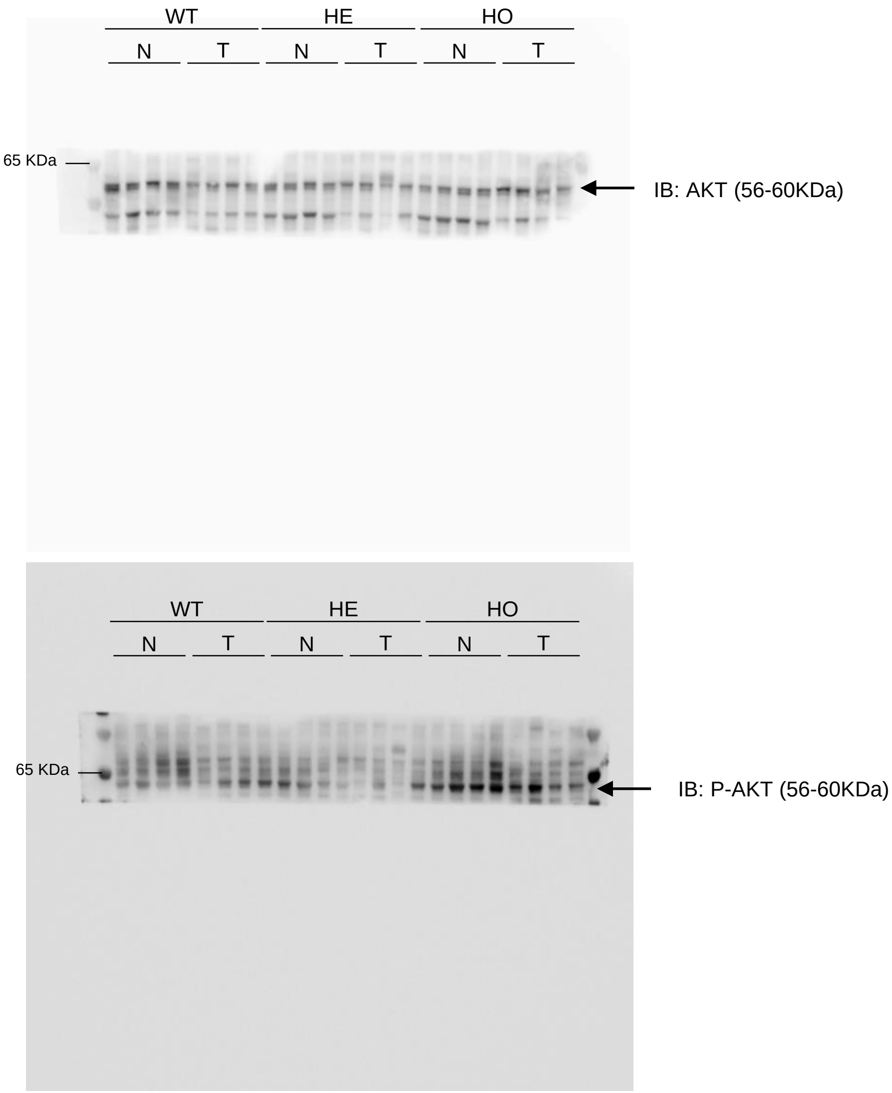

Figure S7. Uncropped blots from Figure 7B. Total AKT, P(Thr308) AKT.

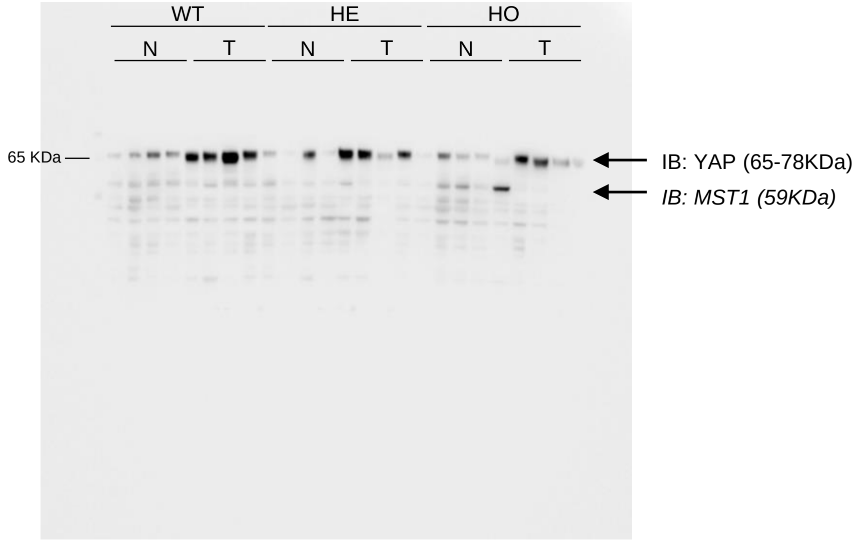

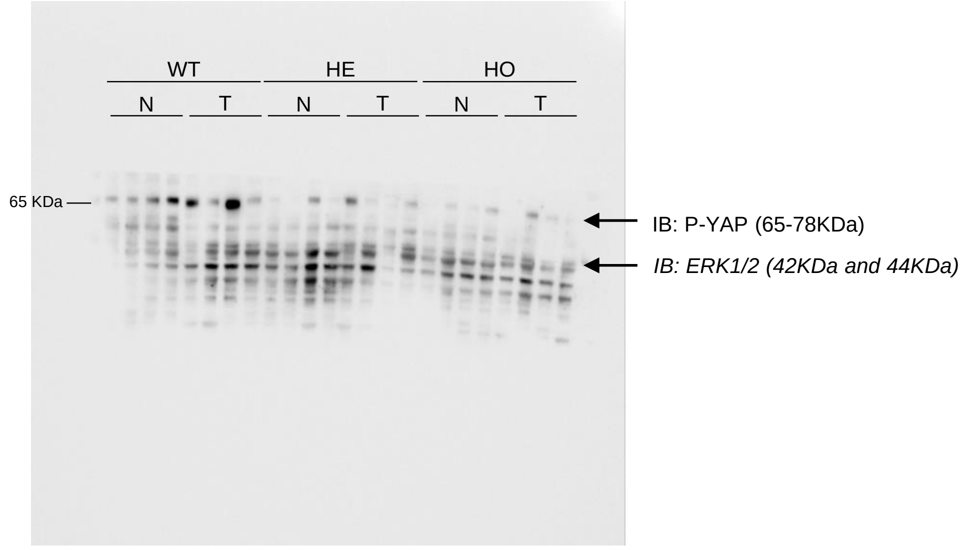

Figure S8. Uncropped blots from Figure 7C. Total YAP, P(Ser127) YAP. Bands corresponding to previous incubations are indicated in italics.

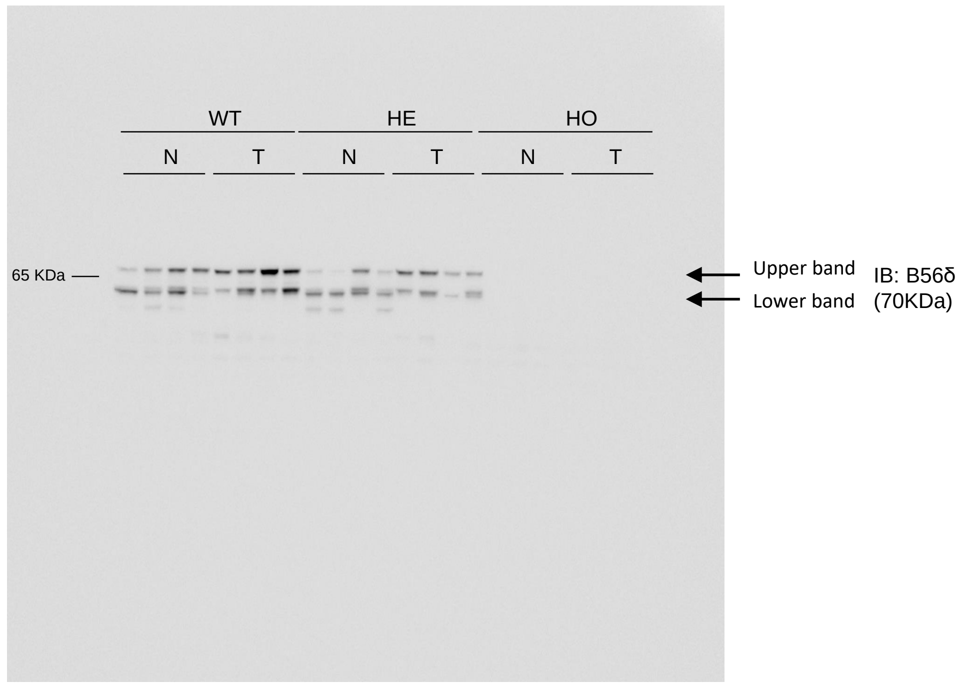

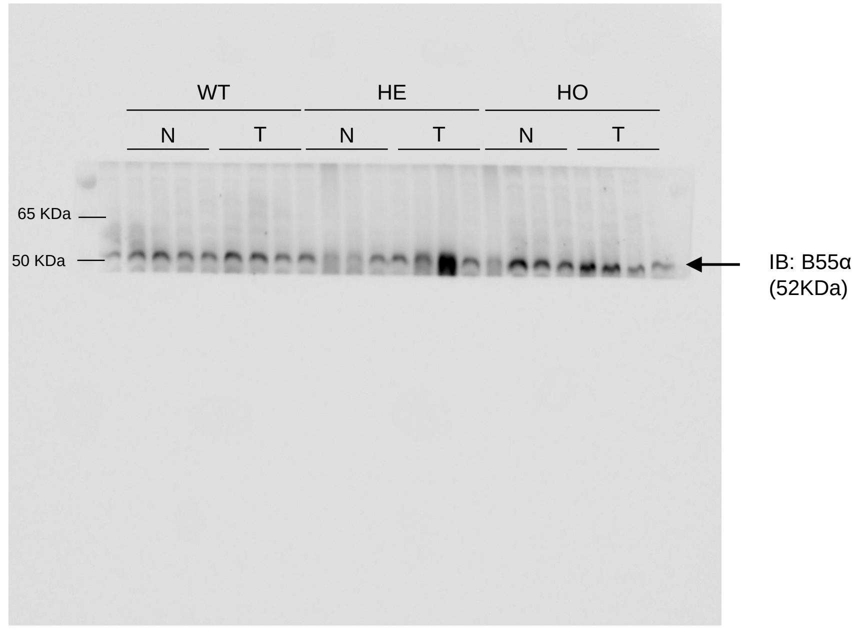

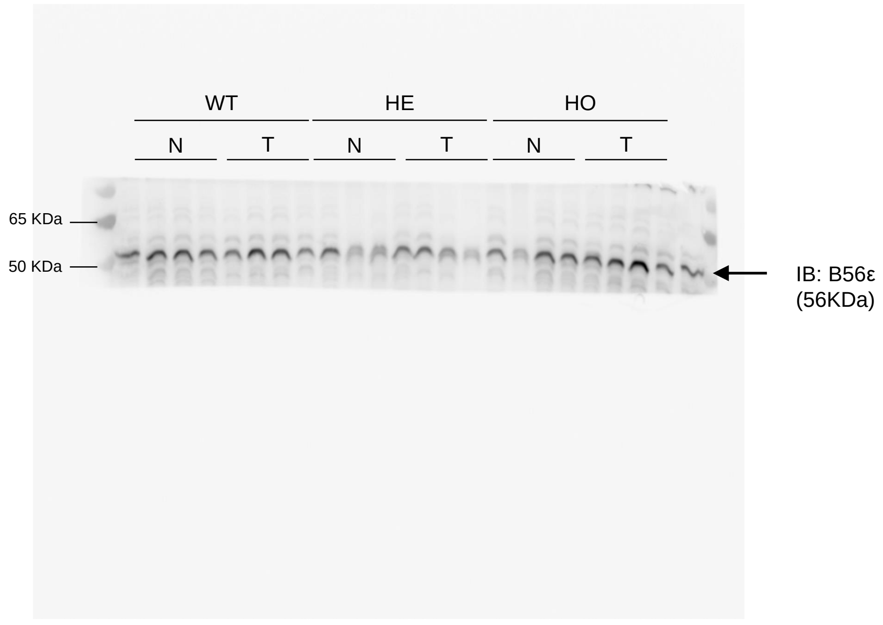

Figure S9: Uncropped blots from Figure 9A, 9B. Total B56δ**,** B55α, B56ε.

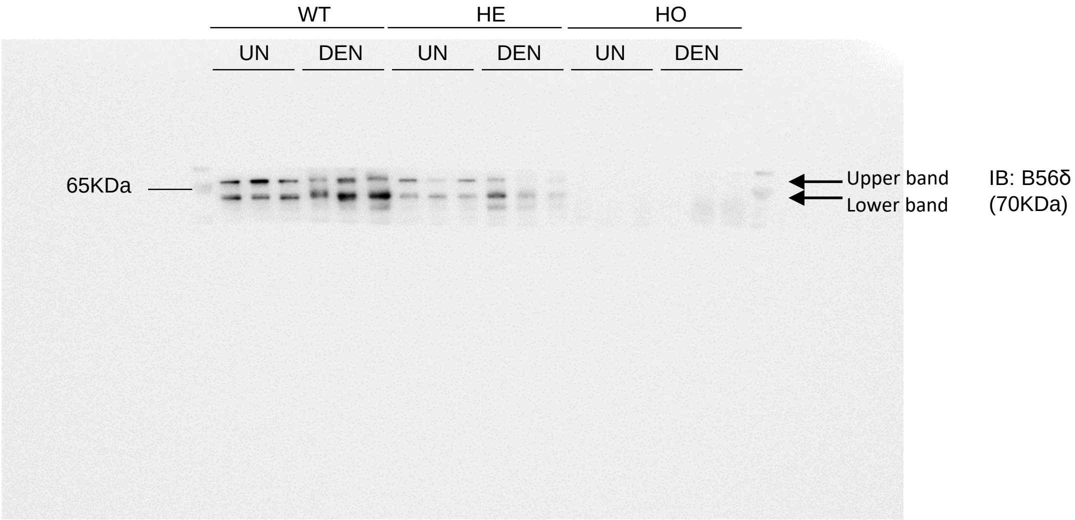

Figure S10. Uncropped blots from Figure 9C. Total B56δ.

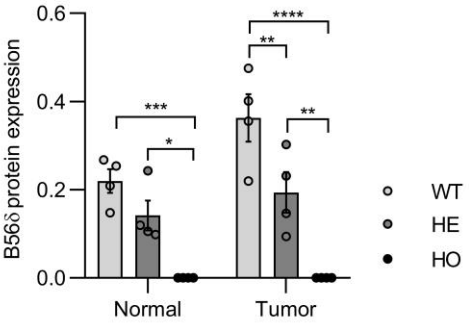

Figure S11. Analysis of B56δ protein expression in WT, HE, and HO *Ppp2r5d* KO mice livers at 11 months post-DEN administration. Protein extracts were prepared from DEN-treated livers of WT, HE, and HO mice. Equal amounts of lysate were separated by SDS-PAGE and subjected to immunoblotting with B56δ antibody. Ponceau was used for normalization. B56δ protein expression was assessed in non-tumor (Normal) and tumor liver (Tumor) tissue of 11 months post-DEN WT, HE, and HO *Ppp2r5d* KO mice. Dots represent individual measurements within a group. Data are represented as mean ± S.E.M. (*p<0.05, **p<0.01, ***p<0.001, ****p<0.0001). Two-way ANOVA analysis was used.

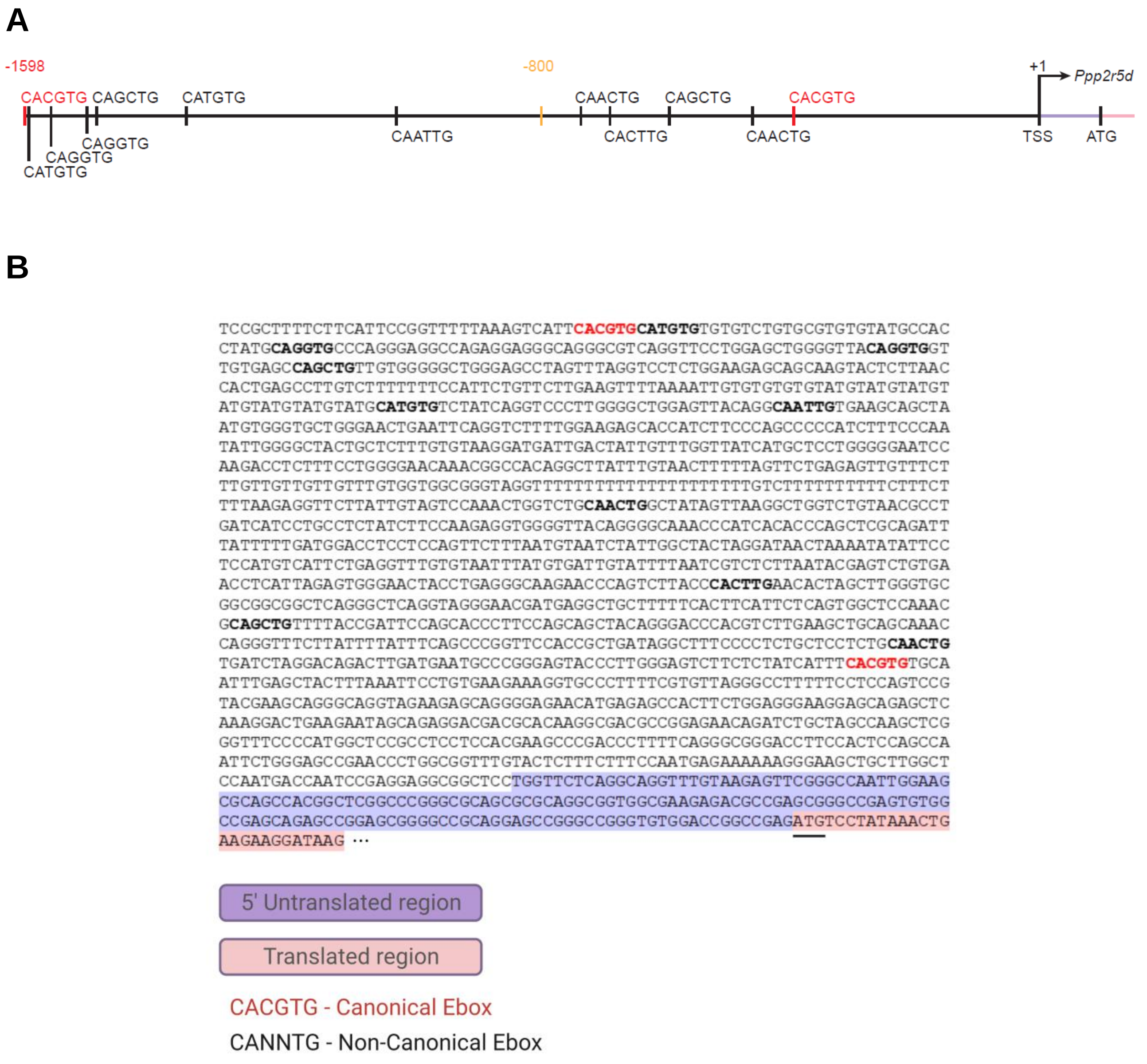

Figure S12. Location of Ebox sequences in the *Ppp2r5d* promotor region of *Mus musculus*. (A) Schematic representation of the consensus Ebox locations in the *Ppp2r5d* promoter region upstream of the transcription start site (TSS). **(B)** Genomic sequence of *Ppp2r5d* promotor. **(A, B)** Canonical Ebox sequences CACGTG (indicated in red) were identified at positions -1604 bp and -388 bp relative to the *Ppp2r5d* TSS. Non-canonical Ebox sequences CANNTG (indicated in black) were identified at positions -1598 bp, -1563 bp, -1506 bp, -1491 bp, -1349 bp, -1017 bp, -725 bp, -679 bp, -585 bp and -454 bp relative to the *Ppp2r5d* TSS. In purple is indicated the 5′ untranslated region, and in pink is the start of the translated region (ATG underlined).
